# Associations of Biomarkers of Inflammation and Breast Cancer in the Breast Adipose Tissue of Women with Combined Measures of Adiposity

**DOI:** 10.1155/2021/3620147

**Published:** 2021-08-13

**Authors:** Sue-Ling Chang, André Tchernof, Francine Durocher, Caroline Diorio

**Affiliations:** ^1^CHU de Québec Research Center, Laval University, Quebec, QC, Canada; ^2^Laval University Cancer Research Center, Quebec, QC, Canada; ^3^School of Nutrition, Faculty of Agriculture and Food Sciences, Laval University, Quebec, QC, Canada; ^4^Quebec Heart Lung Institute, Quebec, QC, Canada; ^5^Department of Molecular Medicine, Faculty of Medicine, Laval University, Quebec, QC, Canada; ^6^Department of Social and Preventive Medicine, Faculty of Medicine, Laval University, Quebec, QC, Canada; ^7^Deschênes-Fabia Center for Breast Diseases, Quebec, QC, Canada

## Abstract

**Background:**

Mechanisms underlying the obesity-breast cancer link involve inflammation but need to be elucidated. Determining obesity by combining body mass index (BMI) with the waist circumference (WC) may clarify the role of inflammatory and hormonally related markers in breast cancer. We examined the effect of combining adiposity indices (BMI/WC) with the gene expression of several biomarkers involved in breast cancer.

**Methods:**

Expression of cytochrome P450 family 19 subfamily A member 1 (CYP19A1), estrogen receptor-alpha (ER-*α*), allograft inflammatory factor 1 (AIF1), cyclooxygenase-2 (COX2), interleukin-6 (IL-6), tumor necrosis factor-alpha (TNF-*α*), and leptin (LEP) in 141 adipose breast tissues was quantified using qPCR method. BMI and WC were measured by a trained nurse and categorized using the median split, BMI^LO^WC^LO^, BMI^LO^WC^HI^, BMI^HI^WC^LO^, and BMI^HI^WC^HI^.

**Results:**

Gene expression of IL-6 (3-fold), TNF-*α* (2-fold), and LEP (2-fold) was higher in the breast adipose tissue of women with high WC regardless of BMI, that is, BMI^LO^WC^HI^ and BMI^HI^WC^HI^ women (all *P* < 0.01). Compared to BMI^LO^WC^LO^ women, gene expression of CYP19A1, COX2, and AIF1 was increased by two-fold in breast adipose tissue of BMI^HI^WC^HI^ women (*P* < 0.10). ER-*α* was not different across adiposity categories.

**Conclusions:**

The expression of some biomarkers, particularly those related to inflammation, is elevated in breast adipose tissue of women with a high WC independent of BMI. Obesity monitoring should also include women with normal or low BMI, but with central adiposity.

## 1. Introduction

Obesity is a complex and serious chronic disease caused by a web of interacting factors (genetic, metabolic, behavioural, and environmental) that are thought to be major drivers behind the continuing rise in obesity prevalence [1]. As obesity numbers climb, so will obesity-related morbidity and mortality [2]. This is especially important because overweight and obesity are strong risk factors for chronic diseases and are causally linked to several cancers, including breast cancer, the most commonly occurring cancer among women [3].

Overweight and obesity are characterized by increased body fatness, often assessed using the body mass index or BMI (weight in kilograms divided by the square of height in meters). However, BMI does not account for body shape or body fat distribution [4–6]. Body fat distribution between individuals with similar BMIs can show significant variation and so may not adequately capture adverse health risks [7]. Research has shown that anthropometric indices such as waist circumference (WC), which approximates the accumulation of abdominal fat, provides additional information on breast cancer risk or prognosis that is independent of BMI [8, 9]. Combining WC with BMI may allow further refinement in identifying individuals at risk for obesity-related health risks [1, 8].

Studies have shown that obesity defined by BMI or WC increases breast cancer risk [9, 10] and mortality [11]. Obesity-related adipose tissue dysfunction in the breast is presumably a major contributing factor in this association [12]. Obesity promotes the expansion and ensuing dysregulation of adipose tissue, which bolsters the production and secretion of adipokines and the infiltration of many immune cell types [13]. These cells produce and release cytokines, notably tumor necrosis factor-*α* and interleukin-6 [12], that induce a state of chronic subclinical inflammation [14]. These cytokines, in turn, activate various pathways that can modify the breast microenvironment into one that promotes tumor initiation and development [12]. Inflamed breast adipose tissue is observed consistently in women with elevated body fat [15]. What is surprising is that adipose tissue expansion in the breast is also found in women with elevated levels of body fat but of normal-weight assessed by BMI [16]. This makes sense when we consider that higher visceral adiposity contributes to systemic and local inflammatory and hormonal imbalances [17] and that breast adipocyte hypertrophy is strongly correlated with WC [18]. Collectively, this means that breast inflammation is telling of a hyperadipose state that appears to be relatively independent of BMI, suggesting that the latter may offer incomplete information, especially when examining relationships in the breast cancer-obesity axis [15]. Given the importance of central obesity, as reflected by the WC, on health outcomes, a few researchers have examined the combined effect of both BMI and WC on breast cancer risk and prognosis with inconsistent but often positive results in increasing risks [19–22].

Here, we sought to understand the effects of combining adiposity indices on the variation in inflammation- and hormone-related markers in the breast microenvironment to shed light on results from prospective studies of breast cancer risk and mortality. By using tissue-based rather than systemic biomarkers, we postulate that we are more likely to measure the local breast tissue-specific effects of obesity on inflammation and provide insight into possible underlying mechanisms. We aimed to investigate the association between obesity, by combining BMI and WC, and the relative gene expression of biomarkers associated with breast cancer in breast adipose tissue.

## 2. Materials and Methods

### 2.1. Study Population

Our study population included pre- and postmenopausal women diagnosed with breast cancer prospectively recruited between January 2011 and May 2012 at the Deschênes-Fabia Center for Breast Diseases in Quebec City, Canada, a breast cancer reference center. Details of the study design, recruitment, and data collection process have been previously published [23]. Briefly, women were invited to participate in the study if they were between 30 and 69 years of age and received no treatment before their mastectomy. In total, 165 women were eligible for the study, which was approved by the CHU de Quebec Research Center Ethics Board. All women provided written consent to participate in this study.

### 2.2. Data Collection

Data concerning breast cancer risk factors such as age at surgery and menopausal status were collected during a telephone interview and anthropometric factors (weight, height, waist circumference, and hip circumference) were measured using standardized methods [24]. BMI was calculated as weight (kg) divided by the squared height (m^2^). Given the small sample size, participants were classified as having either high or low adiposity levels based on the median BMI and WC split. Women with a low BMI <25.5 kg/m^2^ or WC < 86 cm were denoted BMI^LO^ or WC^LO^, while women with a high BMI ≥25.5 kg/m^2^ or WC ≥ 86 cm were denoted BMI^HI^ or WC^HI^. Data were combined to create four categories of joined adiposity parameters: BMI^LO^WC^LO^, BMI^LO^WC^HI^, BMI^HI^WC^LO^, and BMI^HI^WC^HI^.

### 2.3. Quantitative Real-Time PCR Analysis

We used relative gene expression data obtained from a previous study [25]. Briefly, formalin-fixed paraffin-embedded (FFPE) mastectomy specimens were obtained for 141 breast cancer patients. Total RNA was extracted using miRNeasy (FFPE) kit (Qiagen) from fifteen 0.6 mm cores of breast adipose tissue obtained more than 1.0 cm distal to the tumor. For some women, breast adipose could not be harvested due to insufficient material; therefore, the number of women varies between markers. Relative quantity for cytochrome P450 family 19 subfamily A member 1 (CYP19A1), estrogen receptor-alpha (ER*-α*), allograft inflammatory factor 1 isoform 1 (AIF1), cyclooxygenase-2 (COX2), interleukin-6 (IL-6), tumor necrosis factor-alpha (TNF*-α*), and leptin (LEP) was calculated using the fit point method. Relative gene expressions were calculated by applying the delta Ct method [26]. qRT-PCR analyses were performed by the CHU de Quebec Research Center (CHUL) Gene Expression Platform, Quebec, Canada, and were compliant with MIQE guidelines [27, 28].

### 2.4. Statistical Analyses

Descriptive data are presented as means ± standard deviation (SD) or as medians with interquartile ranges (IQRs). Relative CYP19A1, ER-*α*, AIF1, COX2, IL-6, TNF-*α,* and LEP expression was compared across combined adiposity categories (BMI^LO^WC^LO^, BMI^LO^WC^HI^, BMI^HI^WC^LO^, and BMI^HI^WC^HI^) using nonparametric Kruskal–Wallis test for overall group differences. Between-group comparisons were performed with Dunn's post hoc test using Benjamini–Hochberg (BH) false-positive correction [29] using RStudio, version 1.1.143.

Associations between combined adiposity categories and gene expressions were analyzed using linear mixed models with a repeated statement to account for the nonconstant variance among the residuals. All models were adjusted for age at surgery, menopausal status, and PCR batch. Biomarker gene expressions were log-transformed to comply with Gaussian assumptions in the models. Results were back-transformed and expressed as adjusted least-square geometric means and least-square geometric mean ratios with 95% confidence intervals. SAS 9.4 was used to carry out statistical analyses.

### 2.5. Sensitivity Analyses

Sensitivity analyses were carried out after excluding participants with a very low BMI ≤18.5 kg/m^2^ (*n* = 2) since underweight women may differ from the rest of the population [30, 31]. We also conducted an analysis restricting to women diagnosed with higher tumor grade (grades 2 and 3) obtained from medical records to determine associations in a homogenous group (*n* = 95). Finally, we conducted a further sensitivity analysis using standard overweight or obese cut-offs (BMI ≥ 25.0 kg/m^2^ or WC ≥ 88 cm) [32] to examine if the main results still held.

## 3. Results

### 3.1. Patient Characteristics

Table 1 details the characteristics of the 141 study participants across adiposity categories. According to the combined adiposity classification, most women were classified in BMI and WC concordant categories: fifty-nine (41.8%) women were classified BMI^LO^WC^LO^ and 63 (44.7%) BMI^HI^WC^HI^. There were fewer women in BMI and WC discordant categories with ten women (7.1%) classified BMI^LO^WC^HI^ and nine (6.4%) BMI^HI^WC^LO^. Women were older with an average age of 54 years in categories with a high WC than those with a lower WC. Likewise, in these two categories, women were predominantly postmenopausal, 60.0% in BMI^LO^WC^HI^ and 57.1% in BMI^HI^WC^HI^.

### 3.2. Analysis of Relative Gene Expression across Combined Adiposity Categories

**Figure 1:**
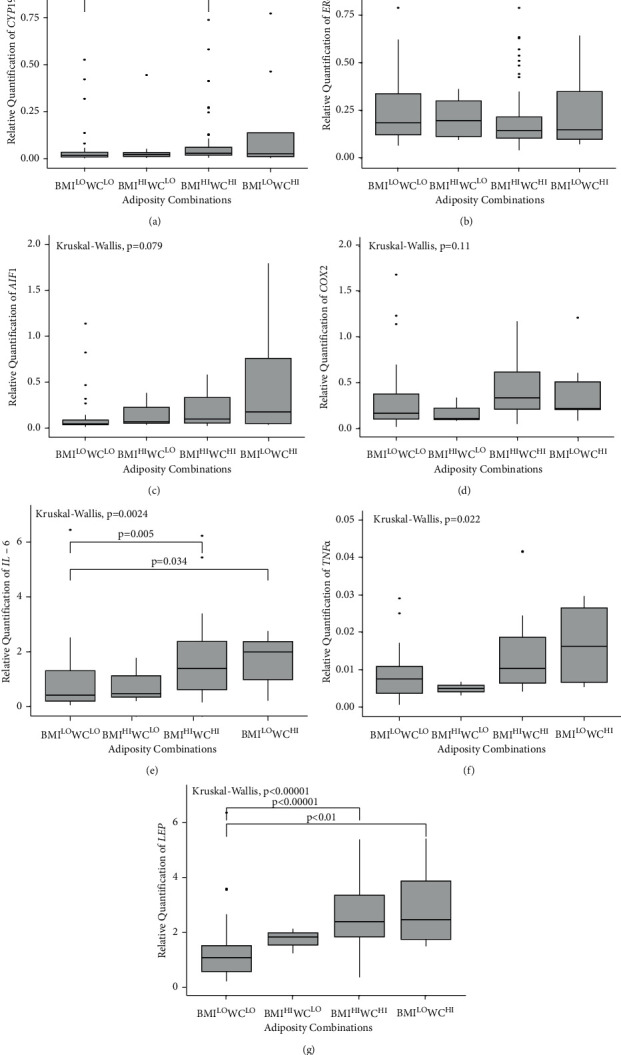
Relative expression of CYP19A1, ER-*α*, AIF1, COX2, IL-6, TNF-*α,* and LEP mRNA in breast adipose tissue, as obtained with RT-qPCR. Box plots represent RQ expression values for each adiposity group. BMI and WC were dichotomized at the median and combined to form the following low (LO) and high (HI) adiposity combined categories: BMI^LO^WC^LO^, BMI^HI^WC^LO^, BMI^HI^WC^HI^, and BMI^LO^WC^HI^. For each adiposity group, biomarker median values are shown as horizontal lines; gray boxes represent the interquartile range and the bars the full range. Kruskal–Wallis test showed significant differences in biomarker gene expression across adiposity groups for (a) CYP19A1 (*P* = 0.017), (e) IL-6 (*P* = 0.0024), (f) TNF*-α* (*P* = 0.022), and (g) LEP (*P* < 0.00001). There were no significant differences in expression of (b) ER*-α* (*P* = 0.42), (c) AIF1 (*P* = 0.079), and (d) COX2 (*P* = 0.11). Significant differences remained after post hoc Dunn's test using Benjamini-Hochberg correction in (a) CYP19A1, BMI^LO^WC^LO^ vs. BMI^HI^WC^HI^, *P* = 0.014, (e) IL-6, BMI^LO^WC^LO^ vs. BMI^HI^WC^HI^, *P* = 0.005, and BMI^LO^WC^LO^ vs. BMI^LO^WC^HI^, *P* = 0.034, and (g) LEP, BMI^LO^WC^LO^ vs. BMI^HI^WC^HI^, *P* < 0.00001, and BMI^LO^WC^LO^ vs. BMI^LO^WC^HI^, *P* < 0.01. RT-PCR = quantitative real-time PCR; RQ = relative quantification; BMI, body mass index; WC = waist circumference.

Analysis of the overall differences in gene expression of CYP19A1, ER-*α*, AIF1, COX2, IL-6, TNF-*α*, and LEP was performed across BMI^LO^WC^LO^, BMI^LO^WC^HI^, BMI^HI^WC^LO^, and BMI^HI^WC^HI^ adiposity categories (Figures 1(a)–1(g)). Gene expression was significantly different overall across adiposity categories for CYP19A1 (*P* = 0.017), IL-6 (*P* = 0.0024), TNF-*α* (*P* = 0.022), and LEP (*P* < 0.00001). There were no overall group differences in expression of ER*-α* (*P* = 0.42) and COX2 (*P* = 0.11), but near significant for AIF1 (*P* = 0.079). When comparing gene expression differences between adiposity groups, BMI^HI^WC^HI^ women had higher CYP19A1 (*P* = 0.014), IL-6 (*P* = 0.005), and LEP expression (*P* < 0.00001) compared to BMI^LO^WC^LO^ women. Expression of IL-6 and LEP was also upregulated in women in the BMI^LO^WC^HI^ category (*P* = 0.034 and *P* < 0.01, respectively) compared to women in the BMI^LO^WC^LO^ category.

### 3.3. Differences in Relative Gene Expression among Combined Adiposity Categories

No significant differences were found in the adjusted geometric means (GM) of CYP19A1, ER-*α*, AIF1, and COX2 across joint adiposity categories (Table 2), although adjusted GMs of CYP19A1, AIF1, and COX2 were borderline significant (*P* = 0.0899, *P* = 0.0633, *P* = 0.0900, respectively). Compared to women in the BMI^LO^WC^LO^ category, mean CYP19A1 expression was significantly higher in BMI^HI^WC^HI^ (ratio of GM (GMR), 1.77; 95% confidence interval (CI), 1.11–2.82; *P* = 0.0184), but not significant in BMI^LO^WC^HI^ women (GMR, 2.53; 95% CI, 0.68–9.39; *P* = 0.1672). Similarly, mean AIF1 expression was higher in BMI^HI^WC^HI^ (GMR, 2.12; 95% CI, 1.17–3.86; *P* = 0.0163) and BMI^LO^WC^HI^ women (GMR, 4.17; 95% CI, 0.93–18.76; *P* = 0.0671). Expression levels of COX2 were also significantly higher in BMI^HI^WC^HI^ (GMR, 2.00; 95%, 1.08–3.69; *P* = 0.0312) but not in BMI^LO^WC^HI^ women (GMR, 2.01; 95%, 0.69–5.90; *P* = 0.2074) when compared to BMI^LO^WC^LO^ women. Adjusted GMs of IL-6, TNF-*α*, and LEP were significantly different across combined categories (all *P* < 0.01) (Table 2). Notably, the expression of these three markers was higher in adiposity categories where WC was high, regardless of BMI. Adjusted mean IL-6 expression levels were higher in BMI^HI^WC^HI^ (GMR, 2.96; 95% CI, 1.59–5.49; *P* = 0.0010) and BMI^LO^WC^HI^ (GMR, 3.21; 95% CI, 1.12–9.17; *P* = 0.0333) compared to BMI^LO^WC^LO^ women. Similarly, GMs of TNF*-α* were higher in BMI^HI^WC^HI^ (GMR, 2.49; 95% CI, 1.55–4.01; *P* = 0.0004) and BMI^LO^WC^HI^ women (GMR, 3.18; 95% CI, 1.06–9.55; *P* = 0.0436). Concerning LEP, GMs were higher in all combinations of BMI and WC when compared to BMI^LO^WC^LO^ women. Compared to women in the BMI^LO^WC^LO^ category, GM ratios were higher in BMI^HI^WC^LO^ (GMR, 1.73; 95% CI, 1.11–2.68; *P* = 0.0176), BMI^HI^WC^HI^ (GMR, 2.49; 95% CI, 1.69–3.68; *P* < 0.0001), and BMI^LO^WC^HI^ women (GMR, 2.31; 95% CI, 1.31–4.08; *P* = 0.0052).

There were no appreciable differences in sensitivity analysis when excluding women with BMI ≤18.5 kg/m^2^. Similar results were obtained when restricted to women diagnosed with higher-grade tumors but were somewhat stronger than those that included lower grades. Analyses using BMI ≥25.0 kg/m^2^ or WC ≥ 88 cm cut-offs showed similar trends, although results for BMI^LO^WC^HI^ were less precise (see Tables S1–S3 in the Supplementary Materials for results of sensitivity analyses).

## 4. Discussion

In this first cross-sectional study, examining breast tissue-specific biomarker expression with combined obesity parameters, we found that mean expression levels of IL-6, TNF*-α,* and LEP were higher in the breast adipose tissue of women with WC ≥ 86 cm, regardless of BMI. Mean expression levels of CYP19A1 and AIF1 were also increased in these women though not significantly in BMI^LO^WC^HI^ women. These results suggest that breast adipose inflammation occurs with excess central adiposity, as proxied here by WC.

Findings from this study share similarities to those reporting systemic or local levels of inflammatory or hormonal dysfunction in normal-weight women as defined by BMI. Inflammation, characterized by crown-like structures, was reportedly present in the breast adipose tissue of 39% of the 72 women with normal-weight (BMI < 25 kg/m^2^) undergoing risk-reducing or treatment mastectomies [16]. What is perhaps more important is that these alterations in breast adipose tissue were similar to those associated with elevated BMI [16]. Specifically, we found that mean expressions of IL-6 (3-fold), TNF*-α* (2-fold), and LEP (2-fold) were higher in the breast adipose tissue of women with high WC regardless of BMI. These findings echo those studies using circulating measurements of inflammatory markers and other adiposity measures, such as those that are correlated with WC. For instance, one study showed that normal-weight women with higher fat mass (FM), defined as BMI < 25 kg/m^2^, and % FM > 30%, had higher levels of circulating proinflammatory cytokines compared to women who are nonobese (*P* < 0.001, for IL-6 and TNF-*α*) [14]. More importantly, these levels were not significantly different from those of women with obesity. Another study of normal-weight postmenopausal women (BMI = 18.5 to 24.9 kg/m^2^) [15] showed that circulating IL-6 and leptin concentrations were significantly higher in women in the highest quartile of trunk fat mass (determined by dual-energy X-ray absorptiometry (DXA)) than in the lowest (*P* < 0.05 for IL-6 and *P* < 0.01 for leptin). Notably, in our study, leptin was significantly expressed in the breast adipose tissue of women with either high BMI or WC compared to BMI^LO^WC^LO^ women. Possibly, because leptin is correlated with overall weight [17], it may be associated with general obesity despite the absence of central adiposity.

Although CYP19A1, AIF1, and COX2 expressions were not significantly different in BMI^LO^WC^HI^ women (possibly due to a lack of power), they were still 2- and 4-fold higher than in BMI^LO^WC^LO^ women. These biomarkers were also elevated in BMI^HI^WC^HI^ women. Still, plausibility is supported by the fact that AIF1 is an adipokine previously described as being correlated with the waist-to-hip ratio (*r* = 0.23) and not BMI [25] and is associated with adverse metabolic phenotypes in women with obesity [33]. Because obesity is associated with increased aromatase activity [34], it is not surprising that women with high WC had increased CYP19A1 expression in breast adipose tissue. We can tie these results to WC because white adipose tissue, in conditions of obesity, contributes to elevated circulating estrogens [35] and subsequent aromatization of the breast adipose tissue [17]. While COX2 mRNA levels have also been higher in the breast tissue of obese than in normal-weight women, it is unclear if this is the case in breast adipose [36]. Unlike the other biomarkers, ER*-α* expression was not significantly different between adiposity categories. Others have reported that ER-*α* is reduced in the abdominal subcutaneous adipose tissue of obese vs. nonobese premenopausal women [37, 38]. *In vitro* studies also suggest that the cancerogenic effects of breast adipocytes are perhaps stimulated by ER-*α* deficiency and that this loss in ER-*α* signaling in adipose tissue promotes obesity [37]. Overall, the fact that biomarker expression in the breast adipose tissue of BMI^HI^WC^LO^ and BMI^LO^WC^LO^ women was similar in our study underscores the importance of central obesity. These findings build from earlier work by Vaysse et al. [39], suggesting that breast adipose tissue inflammation is more likely to occur in patients with visceral adiposity.

To our knowledge, this is the first study examining the expression of inflammatory and hormonal biomarkers in the local breast microenvironment as a function of combined adiposity measurements. How these results tie into those of prospective studies in terms of breast cancer outcomes is yet unclear, and few studies have examined combined adiposity categories with breast cancer risk [19, 22] or prognosis [20, 21]. Notwithstanding methodological differences (anthropometric measurement cut-offs, populations) between the studies, some results are worth highlighting. For instance, Park et al. [19] reported that postmenopausal normal-weight women with central adiposity had more than 40–60% increased risk of developing breast cancer depending on waist size (WC ≥ 80 cm; hazard ratio (HR), 1.38; 95% CI, 1.09–1.75; and WC ≥ 88 cm; HR, 1.58; 95% CI, 1.02–2.46) compared to normal-weight women with low WC [19]. These increased risks were also reported in women with BMI ≥25 kg/m^2^ and central adiposity. Wang et al. [22], on the other hand, did not report significant results in a population of Chinese women using combined WC (≤73.33, 73.33–76.67, 76.67–83.33, and >83.33 cm) and BMI (≤24 and >24 kg/m^2^) cut-offs, although the results for women with BMI ≤24 kg/m^2^ and WC > 83.33 cm were borderline (odds ratio, 1.49; 95% CI, 0.91–2.42). In terms of prognosis, one study found that the risk of breast cancer recurrence and mortality was significantly increased only among women with high BMI (≥25 kg/m^2^) and WC (≥80 cm) or when combined with high breast volume [21]. However, combined adiposity categories had few events, and the authors noted possible insufficient power. Chen et al. [20] reported that combined high WC and high BMI were independent prognostic factors for disease-free and overall survival among women with triple-negative breast cancer. However, this study only compared the results against WC < 80 cm and BMI <25 kg/m^2^ women and discordant adiposity categories were not considered.

Although studies on breast cancer risk and prognosis and combined WC and BMI are divided and few, there is supporting evidence from related studies that underscore the importance of using measurements of central obesity in addition to BMI for risk-stratification. We know from studies of normal-weight postmenopausal women that those with central obesity have a 20% increased risk of dying from all-cause cancer than women without central obesity [6]. In one study using DXA measurements to quantify body fat, normal-weight postmenopausal women in the highest quartile of trunk fat mass had increased breast cancer risk compared to the lowest quartile (HR, 1.88; 95% CI, 1.18–2.98) [15]. WC was positively correlated to trunk fat mass in the study (*r* = 0.68) [15]. Collectively, these findings make sense considering that obesity promotes white adipose tissue dysfunction [40], which then orchestrates many cellular responses: increased secretion of proinflammatory mediators such as TNF-*α* and IL-6 [41], overproduction of leptin by adipocytes [35], and over activation of transcription factors [15, 17, 35]. These biologically active factors are thought to mediate the obesity and cancer relationship [41] by deregulating common signaling pathways, including PI3K/Akt/mTOR, a major pathway implicated in breast cancer and involved in tumor initiation, progression, growth, proliferation, invasion, and metabolism [35]. In women with obesity, systemic and local dysregulation of the inflammatory response is found mainly in visceral fat and breast adipose tissue [41, 42]. Obesity may also trigger inflammatory pathways that increase TNF-*α* production in the adipose tissue, which in turn induce aromatase expression in adipose fibroblasts [34]. Aromatase expressed in adipose tissue could allow for a permissive carcinogenic environment due to the proximity of breast adipocytes to nearby breast epithelial cells [41, 43]. Excessive accumulation of adipose tissue, particularly visceral adipose tissue, may stimulate many local and systemic procarcinogenic mechanisms [40]. These observations support our findings of locally elevated expression levels of inflammatory and hormonal biomarkers in breast adipose tissue in women with high WC, irrespective of their BMI.

Our results, albeit exploratory, suggest important implications from a clinical and public health standpoint. One of the challenges when classifying patients as a function of both BMI and WC is that both these measures are correlated, although not perfectly [44]. This leads to categories of adiposity that contain few women, as was the case in this study and others. However, given that the prevalence of normal-weight metabolically obese individuals is estimated to range between 13% and 38% [45], the proportion of women in discordant adiposity categories in the population, BMI^LO^WC^HI^ or BMI^HI^WC^LO^, is not negligible. What is more, WC seems to be increasing beyond changes expected in BMI [8]. For instance, in Canada, it is estimated that between 1981 and 2007, WC in women increased by 4.9 cm for a BMI of 25 kg/m^2^ [8]. This means that the number of women in these categories stands to increase. There is currently no consensus on which anthropometric measurement is best to assess abdominal adiposity [8]. For its simplicity and clinical applicability, the ICCR Working Group on Visceral Obesity recommended the use of WC [8]. In their consensus statement, they argued for the usage of a combination of BMI and WC to identify high-risk obesity profiles [8]. Considering that normal-weight women, as defined by BMI, carrying excess abdominal fat are currently not flagged for weight-managing interventions, this represents a missed opportunity for risk evaluation and intervention programs for an at-risk group of women [6]. Weaving together the results from this study and those of prospective studies, women with high WC but with normal BMI may share similar inflammatory profiles in the breast adipose tissue as women considered obese by BMI, which could also account for their similar breast cancer risks and prognosis trajectory.

### 4.1. Strengths and Limitations of the Study

Most epidemiologic studies of inflammation, obesity, and breast cancer have used systemic inflammatory markers, but these have short half-lives and are not likely to reflect the effects of obesity directly on the breast microenvironment [12]. Here, we show results for several biomarkers that were quantified in breast adipose tissue, not in close proximity, yet near the breast tumor, thus adding to their relevance. Because previous studies on *in situ* markers of breast tissue inflammation have been mainly limited to crown-like structures [39], our study adds to the body of knowledge on breast tissue inflammation. However, our results should be considered in light of some limitations. We acknowledge that this study involved a small sample size and that consequently discordant adiposity categories were sparse. With this in mind, adiposity categories were created by dichotomizing WC and BMI using the median split, which may have led to misclassification of our obesity exposure. However, our median BMI cut-off of 25.5 kg/m^2^ was within the overweight/obesity range, and our WC of 86 cm falls within the range of what is considered a risky or extremely risky WC (80–88 cm) [32]. As part of our sensitivity analyses, we repeated the analyses using standard cut-offs for obesity (BMI ≥ 25.0 kg/m^2^ or WC ≥ 88 cm) [32], which resulted in smaller sample sizes and larger standard errors for BMI^LO^WC^HI^ women. However, the overall pattern of results remained similar to our main analysis. Although we adjusted for age, menopausal status, and PCR batch, there may be residual confounding from unmeasured confounders. Furthermore, we were unable to explore the effect of menopausal status on associations. Given that mechanisms linking obesity to breast cancer risk may differ between premenopausal and postmenopausal women [46], this question is worth exploring further. Lastly, though our cross-sectional study design prevents the possibility of causal interpretation, it does provide some future lines of questioning.

## 5. Conclusions

We found that CYP19A1, AIF1, IL-6, TNF*-α,* and LEP expression in breast adipose tissue was higher in women with central adiposity, irrespective of BMI. BMI^HI^WC^HI^ and BMI^LO^WC^HI^ women had comparable inflammation- and hormone-related expression profiles in the breast adipose tissue. Considering that central obesity promotes chronic systemic and local inflammation and hormonal imbalances associated with breast cancer risk and poor prognosis, our results imply that an important subgroup of women may be misclassified as “not at risk” if we only use BMI to define obesity. From a public health perspective, this could have many implications as BMI^LO^WC^HI^ women are not currently targeted for risk-reducing strategies. However, further studies are warranted in larger prospective studies to confirm the results of this first study.

## Figures and Tables

**Table 1:** Characteristics of study population *n* = 141.

Characteristics	Adiposity combinations^1^			
	BMI^LO^WC^LO^	BMI^LO^WC^HI^	BMI^HI^WC^LO^	BMI^HI^WC^HI^
*n* (%)	59 (41.8)	10 (7.1)	9 (6.4)	63 (44.7)
Age at surgery, years (mean ± SD)	50.4 ± 7.4	54.4 ± 9.8	52.0 ± 6.9	54.1 ± 7.4
Postmenopausal, *n* (%)	22 (37.3)	6 (60.0)	3 (33.3)	36 (57.1)
BMI, kg/m^2^ (median (IQR))	22.7 (21.2–24.2)	24.3 (23.4–25.2)	26.1 (26.0–26.9)	30.1 (27.4–35.1)
WC, cm (median (IQR))	75.0 (72.0–79.0)	86.0 (86.0–88.0)	83.0 (81.0–85.0)	95.0 (90.0–104.0)

^1^BMI^HI^ is ≥median (25.5 kg/m^2^); WC^HI^ is ≥ median (86.0 cm). BMI = body mass index; WC = waist circumference; LO = low adiposity; HI = high adiposity; SD = standard deviation; IQR = interquartile range.

**Table 2:** Breast adipose tissue biomarker mRNA expression according to combined adiposity (*n* = 141).

Biomarkers		*n*	Geometric means^a^		*P* value ^b^	Ratio^c^		*P* value ^b^
			(95% Cl)			(95% CI)		
CYP19A1	BMI^LO^WC^LO^	59	0.022	(0.016–0.032)	0.0899	1	Ref.	
	BMI^HI^WC^LO^	9	0.027	(0.011–0.066)		1.22	(0.46–3.21)	0.6904
	BMI^LO^WC^HI^	10	0.056	(0.016–0.199)		2.53	(0.68–9.39)	0.1672
	BMI^HI^WC^HI^	63	0.039	(0.029–0.053)		1.77	(1.11–2.82)	**0.0184**
ER*-α*	BMI^LO^WC^LO^	59	0.216	(0.181–0.258)	0.3641	1	Ref.	
	BMI^HI^WC^LO^	9	0.199	(0.137–0.289)		0.92	(0.61–1.39)	0.6932
	BMI^LO^WC^HI^	10	0.185	(0.110–0.313)		0.86	(0.49–1.49)	0.5879
	BMI^HI^WC^HI^	63	0.171	(0.142–0.205)		0.79	(0.61–1.02)	0.0777
AIF1	BMI^LO^WC^LO^	35	0.068	(0.044–0.107)	0.0633	1	Ref.	
	BMI^HI^WC^LO^	3	0.129	(0.024–0.705)		1.89	(0.33–10.80)	0.4768
	BMI^LO^WC^HI^	7	0.285	(0.069–1.180)		4.17	(0.93–18.76)	0.0671
	BMI^HI^WC^HI^	29	0.145	(0.100–0.210)		2.12	(1.17–3.86)	**0.0163**
COX2	BMI^LO^WC^LO^	34	0.192	(0.122–0.300)	0.0900	1	Ref.	
	BMI^HI^WC^LO^	3	0.144	(0.060–0.344)		0.75	(0.29–1.96)	0.5619
	BMI^LO^WC^HI^	7	0.386	(0.149–0.999)		2.01	(0.69–5.90)	0.2074
	BMI^HI^WC^HI^	29	0.383	(0.259–0.566)		2.00	(1.08–3.69)	**0.0312**
IL-6	BMI^LO^WC^LO^	35	0.464	(0.300–0.719)	**0.0085**	1	Ref.	
	BMI^HI^WC^LO^	3	0.654	(0.183–2.343)		1.41	(0.37–5.35)	0.6162
	BMI^LO^WC^HI^	7	1.489	(0.589–3.764)		3.21	(1.12–9.17)	**0.0333**
	BMI^HI^WC^HI^	29	1.373	(0.915–2.060)		2.96	(1.59–5.49)	**0.0010**
TNF*-α*	BMI^LO^WC^LO^	31	0.006	(0.005–0.009)	**0.0006**	1	Ref.	
	BMI^HI^WC^LO^	2	0.004	(0.002–0.008)		0.67	(0.32–1.40)	0.2917
	BMI^LO^WC^HI^	5	0.020	(0.007–0.060)		3.18	(1.06–9.55)	**0.0436**
	BMI^HI^WC^HI^	26	0.016	(0.011–0.022)		2.49	(1.55–4.01)	**0.0004**
LEP	BMI^LO^WC^LO^	35	1.005	(0.766–1.319)	**0.0002**	1	Ref.	
	BMI^HI^WC^LO^	3	1.734	(1.204–2.497)		1.73	(1.11–2.68)	**0.0176**
	BMI^LO^WC^HI^	7	2.323	(1.440–3.747)		2.31	(1.31–4.08)	**0.0052**
	BMI^HI^WC^HI^	29	2.502	(1.927–3.250)		2.49	(1.69–3.68)	**<0.0001**

CYP19A1 = cytochrome P450 family 19 subfamily A member 1; ER*-α* = estrogen receptor-alpha; AIF1 = allograft inflammatory factor 1; COX2 *=* cyclooxygenase-2; IL-6 *=* interleukin-6; TNF*-α* = tumor necrosis factor-alpha; LEP = leptin. ^a^Back-transformed least-square means and confidence intervals (CI) from mixed-effects model performed on natural log-transformed values, adjusted for age at surgery, menopausal status, and PCR batch.^b^*P* values were calculated with mixed models performed on the logarithms of biomarker level data. *P* values in bold indicate *P* < 0.05. ^c^Least-square geometric mean ratio compared with adiposity category BMI^LO^WC^LO^ (reference) after adjusting for age, menopausal status, and PCR batch.

## Data Availability

The data used to support the findings of this study are available from the corresponding author upon request.

## Supplementary Materials

Supplementary Materials Supplementary Table S1: breast adipose tissue biomarker mRNA expression according to combined adiposity in women with BMI >18.5 kg/m^2^ (*n* = 139). Supplementary Table S2: adipose breast tissue biomarker mRNA expression according to combined adiposity in women with grade II/III tumors (*n* = 95). Supplementary Table S3: breast adipose tissue biomarker mRNA expression according to combined adiposity using standard cut-offs (*n* = 141).

## References

[1] Wharton S., Lau D. C. W., Vallis M. (2020). Obesity in adults: a clinical practice guideline. *Canadian Medical Association Journal*.

[2] De Lorenzo A., Gratteri S., Gualtieri P., Cammarano A., Bertucci P., Di Renzo L. (2019). Why primary obesity is a disease?. *Journal of Translational Medicine*.

[3] World Cancer Research Fund/American Institute for Cancer Research (2018). *Continuous Project Report 2018. Diet, Nutrition and Physical Activity: Energy Balance and Body Fatness*.

[4] Pimentel I., Lohmann A. E., Goodwin P. J. (2019). Normal weight Adiposity and postmenopausal breast cancer risk. *JAMA Oncology*.

[5] Brown J. C., Cespedes Feliciano E. M., Caan B. J. (2018). The evolution of body composition in oncology-epidemiology, clinical trials, and the future of patient care: facts and numbers. *Journal of Cachexia, Sarcopenia and Muscle*.

[6] Sun Y., Liu B., Snetselaar L. G. (2019). Association of normal-weight central obesity with all-cause and cause-specific mortality among postmenopausal women. *JAMA Network Open*.

[7] Tchernof A., Després J.-P. (2013). Pathophysiology of human visceral obesity: an update. *Physiological Reviews*.

[8] Ross R., Neeland I. J., Yamashita S. (2020). Waist circumference as a vital sign in clinical practice: a consensus statement from the IAS and ICCR working group on visceral obesity. *Nature Reviews Endocrinology*.

[9] Barberio A. M., Alareeki A., Viner B. (2019). Central body fatness is a stronger predictor of cancer risk than overall body size. *Nature Communications*.

[10] Xia X., Chen W., Li J. (2014). Body mass index and risk of breast cancer: a nonlinear dose-response meta-analysis of prospective studies. *Scientific Reports*.

[11] Protani M., Coory M., Martin J. H. (2010). Effect of obesity on survival of women with breast cancer: systematic review and meta-analysis. *Breast Cancer Research and Treatment*.

[12] Matthews S. B., Thompson H. J. (2016). The obesity-breast cancer conundrum: an analysis of the issues. *International Journal of Molecular Sciences*.

[13] Vijay J., Gauthier M.-F., Biswell R. L. (2020). Single-cell analysis of human adipose tissue identifies depot- and disease-specific cell types. *Nature Metabolism*.

[14] De Lorenzo A., Del Gobbo V., Premrov M. G., Bigioni M., Galvano F., Di Renzo L. (2007). Normal-weight obese syndrome: early inflammation?. *American Journal of Clinical Nutrition*.

[15] Iyengar N. M., Arthur R., Manson J. E. (2019). Association of body fat and risk of breast cancer in postmenopausal women with normal body mass index. *JAMA Oncology*.

[16] Iyengar N. M., Brown K. A., Zhou X. K. (2017). Metabolic obesity, adipose inflammation and elevated breast aromatase in women with normal body mass index. *Cancer Prevention Research*.

[17] Zimta A.-A., Tigu A. B., Muntean M., Cenariu D., Slaby O., Berindan-Neagoe I. (2019). Molecular links between central obesity and breast cancer. *International Journal of Molecular Sciences*.

[18] Laforest S., Durocher F., Tchernof A., Diorio C. (2015). Breast adipocyte hypertrophy is associated with a high waist circumference independent of body mass index in women with breast cancer. *CMR eJournal*.

[19] Park Y. M. M., White A. J., Nichols H. B., O’Brien K. M., Weinberg C. R., Sandler D. P. (2017). The association between metabolic health, obesity phenotype and the risk of breast cancer. *International Journal of Cancer*.

[20] Chen H. L., Ding A., Wang M. L. (2016). Impact of central obesity on prognostic outcome of triple negative breast cancer in Chinese women. *SpringerPlus*.

[21] Wisse A., Tryggvadottir H., Simonsson M. (2018). Increasing preoperative body size in breast cancer patients between 2002 and 2016: implications for prognosis. *Cancer Causes & Control*.

[22] Wang F., Liu L., Cui S. (2017). Distinct effects of body mass index and waist/hip ratio on risk of breast cancer by joint estrogen and progestogen receptor status: results from a case‐control study in northern and eastern China and implications for chemoprevention. *The Oncologist*.

[23] Hanna M., Dumas I., Jacob S., Têtu B., Diorio C. (2015). Physical activity, mammographic density, and age-related lobular involution among premenopausal and postmenopausal women. *Menopause*.

[24] US Department of Health and Human Services, National Center for Health Statistics (1996). *The Third National Health and Nutrition Examination Survey (NHANES III, 1988–1994)*.

[25] Slim F. A., Ouellette G., Ennour-Idrissi K., Jacob S., Diorio C., Durocher F. (2018). An isoform of AIF1 involved in breast cancer. *Cancer Cell International*.

[26] Pfaffl M. W. (2001). A new mathematical model for relative quantification in real-time RT–PCR. *Nucleic Acids Research*.

[27] Bustin S. A., Benes V., Garson J. A. (2009). *The MIQE Guidelines: Minimum Information for Publication of Quantitative Real-Time PCR Experiments*.

[28] Bustin S. A., Beaulieu J.-F., Huggett J. (2010). *MIQE Precis: Practical Implementation of Minimum Standard Guidelines for Fluorescence-Based Quantitative Real-Time PCR Experiments*.

[29] Benjamini Y., Hochberg Y. (1995). Controlling the false discovery rate: a practical and powerful approach to multiple testing. *Journal of the Royal Statistical Society: Series B*.

[30] Oudanonh T., Nabi H., Ennour‐Idrissi K., Lemieux J., Diorio C. (2020). Progesterone receptor status modifies the association between body mass index and prognosis in women diagnosed with estrogen receptor positive breast cancer. *International Journal of Cancer*.

[31] Nabi H., Diorio C. (2018). Body mass index and clinical outcomes in trastuzumab-treated metastatic breast cancer patients: an alternative explanation for the lack of association. *The Breast*.

[32] (2000). Obesity: preventing and managing the global epidemic. Report of a WHO consultation. *World Health Organization Technical Report Series*.

[33] Lorente-Cebrián S., Decaunes P., Dungner E., Bouloumié A., Arner P., Dahlman I. (2013). Allograft inflammatory factor 1 (AIF-1) is a new human adipokine involved in adipose inflammation in obese women. *BMC Endocrine Disorders*.

[34] Bulun S. E., Chen D., Moy I., Brooks D. C., Zhao H. (2012). Aromatase, breast cancer and obesity: a complex interaction. *Trends in Endocrinology and Metabolism*.

[35] Wang X., Simpson E. R., Brown K. A. (2015). Aromatase overexpression in dysfunctional adipose tissue links obesity to postmenopausal breast cancer. *The Journal of Steroid Biochemistry and Molecular Biology*.

[36] Bowers L. W., deGraffenried L. A. (2015). Targeting the COX-2 pathway to improve therapeutic response in the obese breast cancer patient population. *Current Pharmacology Reports*.

[37] Drew B. G., Hamidi H., Zhou Z. (2015). Estrogen receptor (ER)*α*-regulated lipocalin 2 expression in adipose tissue links obesity with breast cancer progression. *Journal of Biological Chemistry*.

[38] Nilsson M., Dahlman I., Rydén M. (2007). Oestrogen receptor *α* gene expression levels are reduced in obese compared to normal weight females. *International Journal of Obesity*.

[39] Vaysse C., Lømo J., Garred Ø. (2017). Inflammation of mammary adipose tissue occurs in overweight and obese patients exhibiting early-stage breast cancer. *Npj Breast Cancer*.

[40] Agurs-Collins T., Ross S. A., Dunn B. K. (2019). The many faces of obesity and its influence on breast cancer risk. *Frontiers in Oncology*.

[41] Rybinska I., Agresti R., Trapani A., Tagliabue E., Triulzi T. (2020). Adipocytes in breast cancer, the thick and the thin. *Cells*.

[42] Simone V., D’Avenia M., Argentiero A. (2016). Obesity and breast cancer: molecular interconnections and potential clinical applications. *The Oncologist*.

[43] Kothari C., Diorio C., Durocher F. (2020). The importance of breast adipose tissue in breast cancer. *International Journal of Molecular Sciences*.

[44] Folsom A. R., Kushi L. H., Anderson K. E. (2000). Associations of general and abdominal obesity with multiple health outcomes in older women. *Archives of Internal Medicine*.

[45] Bosomworth N. J. (2019). Normal-weight central obesity. *Canadian Family Physician*.

[46] Yung R. L., Ligibel J. A. (2016). Obesity and breast cancer: risk, outcomes, and future considerations. *Clinical Advances in Hematology & Oncology: Human Organization*.

