# Supplementary material for: Associations of Biomarkers of Inflammation and Breast Cancer in the Breast Adipose Tissue of Women with Combined Measures of Adiposity
Source: J Obes. 2021 Aug 13;2021:3620147. doi: 10.1155/2021/3620147 (PMC8380177; doi:10.1155/2021/3620147)
Supplement: Supplementary Materials — Supplementary Table S1: breast adipose tissue biomarker mRNA expression according to combined adiposity in women with BMI >18.5 kg/m2 (n = 139). Supplementary Table S2: adipose breast tissue biomarker mRNA expression according to combined adiposity in women with grade II/III tumors (n = 95). Supplementary Table S3: breast adipose tissue biomarker mRNA expression according to combined adiposity using standard cut-offs (n = 141). [file 3620147.f1.zip › 3620147.f1/Supplementary Table 1.pdf]

Table S1. Breast Adipose Tissue Biomarker mRNA Expression According to Combined Adiposity in women with BMI > 18.5 kg/m^2 * (N=139)

Biomarkers		n	Geometric Means [a] (95% CI)		*P* value [b]	Ratio [c] (95% CI)		*P* value [b]
CYP19A1	$BMI^{LO}WC^{LO}$	57	0.023	(0.016 - 0.033)	0.1163	1	Ref.	
	$BMI^{HI}WC^{LO}$	9	0.027	(0.011 - 0.066)		1.20	(0.45 - 3.16)	0.7175
	$BMI^{LO}WC^{HI}$	10	0.056	(0.016 - 0.197)		2.47	(0.67 - 9.17)	0.1779
	$BMI^{HI}WC^{HI}$	63	0.039	(0.029 - 0.053)		1.73	(1.08 - 2.79)	**0.0254**
ER-α	$BMI^{LO}WC^{LO}$	57	0.220	(0.184 - 0.263)	0.3121	1	Ref.	
	$BMI^{HI}WC^{LO}$	9	0.199	(0.137 - 0.290)		0.91	(0.60 - 1.38)	0.6499
	$BMI^{LO}WC^{HI}$	10	0.185	(0.110 - 0.311)		0.84	(0.48 - 1.46)	0.5380
	$BMI^{HI}WC^{HI}$	63	0.171	(0.142 - 0.206)		0.78	(0.60 - 1.01)	0.0619
AIF1	$BMI^{LO}WC^{LO}$	33	0.068	(0.043 - 0.109)	0.0719	1	Ref.	
	$BMI^{HI}WC^{LO}$	3	0.129	(0.023 - 0.713)		1.88	(0.32 - 10.96)	0.4857
	$BMI^{LO}WC^{HI}$	7	0.291	(0.071 - 1.193)		4.25	(0.95 - 19.15)	0.0637
	$BMI^{HI}WC^{HI}$	29	0.146	(0.101 - 0.211)		2.13	(1.15 - 3.95)	**0.0191**
COX2	$BMI^{LO}WC^{LO}$	32	0.205	(0.129 - 0.326)	0.1327	1	Ref.	
	$BMI^{HI}WC^{LO}$	3	0.147	(0.061 - 0.351)		0.71	(0.27 - 1.88)	0.4982
	$BMI^{LO}WC^{HI}$	7	0.388	(0.152 - 0.993)		1.89	(0.65 - 5.54)	0.2494
	$BMI^{HI}WC^{HI}$	29	0.383	(0.259 - 0.567)		1.87	(1.00 - 3.50)	0.0559
IL-6	$BMI^{LO}WC^{LO}$	33	0.493	(0.313 - 0.777)	**0.0179**	1	Ref.	
	$BMI^{HI}WC^{LO}$	3	0.665	(0.184 - 2.396)		1.35	(0.35 - 5.17)	0.6654
	$BMI^{LO}WC^{HI}$	7	1.497	(0.599 - 3.738)		3.03	(1.06 - 8.67)	**0.0423**
	$BMI^{HI}WC^{HI}$	29	1.372	(0.916 - 2.057)		2.78	(1.48 - 5.23)	**0.0023**
TNF-α	$BMI^{LO}WC^{LO}$	29	0.006	(0.005 - 0.009)	**0.0012**	1	Ref.	
	$BMI^{HI}WC^{LO}$	2	0.004	(0.002 - 0.008)		0.63	(0.30 - 1.31)	0.2199
	$BMI^{LO}WC^{HI}$	5	0.020	(0.007 - 0.057)		3.00	(1.00 - 8.88)	**0.0540**
	$BMI^{HI}WC^{HI}$	26	0.016	(0.011 - 0.022)		2.32	(1.47 - 3.72)	**0.0010**
LEP	$BMI^{LO}WC^{LO}$	33	1.038	(0.781 - 1.380)	**0.0006**	1	Ref.	
	$BMI^{HI}WC^{LO}$	3	1.750	(1.211 - 2.530)		1.69	(1.08 - 2.64)	**0.0255**
	$BMI^{LO}WC^{HI}$	7	2.338	(1.454 - 3.758)		2.25	(1.27 - 4.00)	**0.0073**
	$BMI^{HI}WC^{HI}$	29	2.509	(1.933 - 3.257)		2.42	(1.62 - 3.61)	**<.0001**

CYP19A1 Cytochrome P450 family 19 subfamily A member 1, *ER-α* Estrogen receptor alpha, *AIF1* Allograft inflammatory factor 1, *COX2* Cyclooxygenase-2, *IL-6* Interleukin 6, *TNF-α* Tumor necrosis factor-alpha, *LEP* Leptin.

[a]Back transformed least-square means and confidence intervals (CI) from mixed-effects model performed on natural log-transformed values. Adjusted for age at surgery, menopausal status and PCR batch.

[b]P values were calculated with mixed models performed on the logarithms of biomarker level data. P values in bold indicate $P < 0.05$.

[c]Least square geometric mean ratio comparing with adiposity category $BMI^{LO}WC^{LO}$ (reference) after adjusting for age, menopausal status and PCR batch.
