# Supplementary material for: Associations of Biomarkers of Inflammation and Breast Cancer in the Breast Adipose Tissue of Women with Combined Measures of Adiposity
Source: J Obes. 2021 Aug 13;2021:3620147. doi: 10.1155/2021/3620147 (PMC8380177; doi:10.1155/2021/3620147)
Supplement: Supplementary Materials — Supplementary Table S1: breast adipose tissue biomarker mRNA expression according to combined adiposity in women with BMI >18.5 kg/m2 (n = 139). Supplementary Table S2: adipose breast tissue biomarker mRNA expression according to combined adiposity in women with grade II/III tumors (n = 95). Supplementary Table S3: breast adipose tissue biomarker mRNA expression according to combined adiposity using standard cut-offs (n = 141). [file 3620147.f1.zip › 3620147.f1/Supplementary Table 2.pdf]

Table S2. Adipose Breast Tissue Biomarker mRNA Expression According to Combined Adiposity in women with grade II/III tumors (N=95)

Biomarkers		n	Geometric Means [a] (95% CI)		*P* value [b]	Ratio [c] (95% CI)		*P* value [b]
CYP19A1	$BMI^{LO}WC^{LO}$	33	0.016	(0.012 - 0.024)	**0.0011**	1	Ref.	
	$BMI^{HI}WC^{LO}$	3	0.017	(0.007 - 0.043)		1.03	(0.37 - 2.85)	0.9537
	$BMI^{LO}WC^{HI}$	7	0.032	(0.007 - 0.149)		1.98	(0.41 - 9.56)	0.3961
	$BMI^{HI}WC^{HI}$	52	0.045	(0.033 - 0.061)		2.74	(1.67 - 4.51)	**0.0001**
ER-α	$BMI^{LO}WC^{LO}$	33	0.185	(0.145 - 0.237)	0.2858	1	Ref.	
	$BMI^{HI}WC^{LO}$	3	0.215	(0.125 - 0.370)		1.16	(0.64 - 2.12)	0.6259
	$BMI^{LO}WC^{HI}$	7	0.125	(0.087 - 0.181)		0.68	(0.44 - 1.05)	0.0861
	$BMI^{HI}WC^{HI}$	52	0.177	(0.143 - 0.219)		0.95	(0.68 - 1.33)	0.7813
AIF1	$BMI^{LO}WC^{LO}$	23	0.048	(0.031 - 0.074)	**0.0003**	1	Ref.	
	$BMI^{HI}WC^{LO}$	0		-			-	
	$BMI^{LO}WC^{HI}$	5	0.230	(0.034 - 1.544)		4.84	(0.67 - 34.73)	0.1241
	$BMI^{HI}WC^{HI}$	21	0.193	(0.127 - 0.296)		4.07	(2.17 - 7.63)	**<.0001**
COX2	$BMI^{LO}WC^{LO}$	22	0.140	(0.081 - 0.243)	0.0786	1	Ref.	
	$BMI^{HI}WC^{LO}$	0		-			-	
	$BMI^{LO}WC^{HI}$	5	0.268	(0.103 - 0.695)		1.91	(0.61 - 5.97)	0.2726
	$BMI^{HI}WC^{HI}$	21	0.335	(0.212 - 0.527)		2.38	(1.15 - 4.96)	**0.0250**
IL-6	$BMI^{LO}WC^{LO}$	23	0.364	(0.213 - 0.622)	**0.005**	1	Ref.	
	BMI^{HI}/WC^{LO}	0		-			-	
	BMI^{LO}/WC^{HI}	5	1.130	(0.387 - 3.306)		3.10	(0.90 - 10.68)	0.0797
	BMI^{HI}/WC^{HI}	21	1.330	(0.826 - 2.141)		3.65	(1.75 - 7.63)	**0.0013**
TNF-α	$BMI^{LO}WC^{LO}$	21	0.005	(0.003 - 0.007)	**0.0015**	1	Ref.	
	$BMI^{HI}WC^{LO}$	0		-			-	
	$BMI^{LO}WC^{HI}$	4	0.019	(0.007 - 0.056)		3.94	(1.22 - 12.71)	**0.0272**
	$BMI^{HI}WC^{HI}$	18	0.015	(0.010 - 0.022)		3.02	(1.70 - 5.36)	**0.0006**
LEP	$BMI^{LO}WC^{LO}$	23	0.978	(0.725 - 1.319)	**0.0003**	1	Ref.	
	$BMI^{HI}WC^{LO}$	0		-			-	
	$BMI^{LO}WC^{HI}$	5	2.131	(1.254 - 3.622)		2.18	(1.15 - 4.12)	**0.0210**
	$BMI^{HI}WC^{HI}$	21	2.793	(1.973 - 3.954)		2.86	(1.78 - 4.58)	**<.0001**

CYP19A1 Cytochrome P450 family 19 subfamily A member 1, *ER-α* Estrogen receptor alpha, *AIF1* Allograft inflammatory factor 1, *COX2* Cyclooxygenase-2, *IL-6* Interleukin 6, *TNF-α* Tumor necrosis factor-alpha, *LEP* Leptin.

[a]Back transformed least-square means and confidence intervals (CI) from mixed-effects model performed on natural log-transformed values. Adjusted for age at surgery, menopausal status and PCR batch.

[b]P values were calculated with mixed models performed on the logarithms of biomarker level data. P values in bold indicate P < 0.05.

[c]Least square geometric mean ratio comparing with adiposity category $BMI^{LO}WC^{LO}$ (reference) after adjusting for age, menopausal status and PCR batch.
