# Supplementary material for: Associations of Biomarkers of Inflammation and Breast Cancer in the Breast Adipose Tissue of Women with Combined Measures of Adiposity
Source: J Obes. 2021 Aug 13;2021:3620147. doi: 10.1155/2021/3620147 (PMC8380177; doi:10.1155/2021/3620147)
Supplement: Supplementary Materials — Supplementary Table S1: breast adipose tissue biomarker mRNA expression according to combined adiposity in women with BMI >18.5 kg/m2 (n = 139). Supplementary Table S2: adipose breast tissue biomarker mRNA expression according to combined adiposity in women with grade II/III tumors (n = 95). Supplementary Table S3: breast adipose tissue biomarker mRNA expression according to combined adiposity using standard cut-offs (n = 141). [file 3620147.f1.zip › 3620147.f1/Supplementary Table 3.pdf]

Table S3. Breast Adipose Tissue Biomarker mRNA Expression According to Combined Adiposity using standard cut-offs (N=141)

Biomarkers		n	Geometric Means [a] (95% CI)		*P* value [b]	Ratio [c] (95% CI)		*P* value [b]
CYP19A1	$BMI^{LO}WC^{LO}$	60	0.025	(0.017 - 0.036)	0.1568	1	Ref.	
	$BMI^{HI}WC^{LO}$	23	0.033	(0.017 - 0.064)		1.33	(0.62 - 2.85)	0.4632
	$BMI^{LO}WC^{HI}$	2	0.202	(0.025 - 1.602)		8.11	(0.98 - 66.85)	0.0538
	$BMI^{HI}WC^{HI}$	56	0.036	(0.027 - 0.048)		1.44	(0.90 - 2.31)	0.1337
ER-α	$BMI^{LO}WC^{LO}$	60	0.221	(0.186 - 0.263)	0.1229	1	Ref.	
	$BMI^{HI}WC^{LO}$	23	0.188	(0.142 - 0.248)		0.85	(0.61 - 1.18)	0.3351
	$BMI^{LO}WC^{HI}$	2	0.337	(0.065 - 1.750)		1.53	(0.29 - 8.01)	0.6185
	$BMI^{HI}WC^{HI}$	56	0.162	(0.133 - 0.196)		0.73	(0.56 - 0.96)	**0.0218**
AIF1	$BMI^{LO}WC^{LO}$	37	0.079	(0.051 - 0.122)	**0.0176**	1	Ref.	
	$BMI^{HI}WC^{LO}$	11	0.133	(0.059 - 0.300)		1.68	(0.67 - 4.24)	0.2742
	$BMI^{LO}WC^{HI}$	1	3.838	(0.326 - 45.239)		48.65	(3.92 - 604.22)	**0.0036**
	$BMI^{HI}WC^{HI}$	25	0.136	(0.091 - 0.203)		1.72	(0.94 - 3.16)	0.0842
COX2	$BMI^{LO}WC^{LO}$	36	0.239	(0.153 - 0.374)	0.2112	1	Ref.	
	$BMI^{HI}WC^{LO}$	11	0.196	(0.096 - 0.400)		0.82	(0.35 - 1.89)	0.6414
	$BMI^{LO}WC^{HI}$	1	0.083	(0.008 - 0.909)		0.35	(0.03 - 4.01)	0.4006
	$BMI^{HI}WC^{HI}$	25	0.374	(0.256 - 0.546)		1.56	(0.86 - 2.84)	0.1492
IL-6	$BMI^{LO}WC^{LO}$	37	0.567	(0.368 - 0.873)	0.1037	1	Ref.	
	$BMI^{HI}WC^{LO}$	11	0.830	(0.433 - 1.588)		1.46	(0.67 - 3.18)	0.3396
	$BMI^{LO}WC^{HI}$	1	2.427	(0.205 - 28.775)		4.28	(0.34 - 53.57)	0.2633
	$BMI^{HI}WC^{HI}$	25	1.273	(0.796 - 2.038)		2.25	(1.17 - 4.31)	**0.0177**
TNF-α	$BMI^{LO}WC^{LO}$	32	0.007	(0.005 - 0.010)	**0.0312**	1	Ref.	
	$BMI^{HI}WC^{LO}$	10	0.011	(0.005 - 0.022)		1.44	(0.64 - 3.23)	0.3814
	$BMI^{LO}WC^{HI}$	0		-		-		
	$BMI^{HI}WC^{HI}$	22	0.014	(0.010 - 0.020)		1.96	(1.21 - 3.19)	**0.0087**
LEP	$BMI^{LO}WC^{LO}$	37	1.083	(0.831 - 1.410)	**0.0009**	1	Ref.	
	$BMI^{HI}WC^{LO}$	11	1.956	(1.489 - 2.571)		1.81	(1.24 - 2.64)	**0.0031**
	$BMI^{LO}WC^{HI}$	1	2.812	(0.632 - 12.509)		2.60	(0.57 - 11.94)	0.2241
	$BMI^{HI}WC^{HI}$	25	2.508	(1.854 - 3.391)		2.32	(1.54 - 3.49)	**0.0001**

CYP19A1 Cytochrome P450 family 19 subfamily A member 1, *ER-α* Estrogen receptor alpha, *AIF1* Allograft inflammatory factor 1, *COX2* Cyclooxygenase-2, *IL-6* Interleukin 6, *TNF-α* Tumor necrosis factor-alpha, *LEP* Leptin.

[a]Back transformed least-square means and confidence intervals (CI) from mixed-effects model performed on natural log-transformed values. Adjusted for age at surgery, menopausal status and PCR batch.

[b]P values were calculated with mixed models performed on the logarithms of biomarker level data. P values in bold indicate $P < 0.05$.

[c]Least square geometric mean ratio comparing with adiposity category $BMI^{LO}WC^{LO}$ (reference) after adjusting for age, menopausal status and PCR batch.
